# A microswitch-aided program to enable people with extensive multiple disabilities to control environmental stimulation through different responses

**DOI:** 10.3389/fpsyt.2022.1073650

**Published:** 2022-12-09

**Authors:** Giulio E. Lancioni, Nirbhay N. Singh, Mark F. O'Reilly, Jeff Sigafoos, Gloria Alberti, Valeria Chiariello, Lorenzo Desideri

**Affiliations:** ^1^Department of Neuroscience and Sense Organs, University of Bari, Bari, Italy; ^2^Department of Psychiatry and Health Behavior, Augusta University, Augusta, GA, United States; ^3^College of Education, University of Texas at Austin, Austin, TX, United States; ^4^School of Education, Victoria University of Wellington, Wellington, New Zealand; ^5^Lega F. D'Oro Research Center, Osimo, Italy; ^6^Department of Psychology, University of Bologna, Bologna, Italy

**Keywords:** technology, microswitches, stimulation, intellectual disability, sensory impairment, motor impairment

## Abstract

**Objectives:**

This study assessed whether a simple technology-aided program (i.e., a program involving the use of microswitches linked to a smartphone) could be set up to enable people with motor, sensory and intellectual disabilities to control preferred environmental stimulation through two different response movements.

**Methods:**

Ten participants were involved in the study. Each of them was exposed to an ABAB design, in which A represented baseline phases without the program and B intervention phases with the use of the program. The study assessed whether the participants (a) had significant increases of each of the two response movements available and/or showed response variability across sessions and over time and (b) had signs of satisfaction/happiness during the study sessions, in connection with their stimulation access and control.

**Results:**

The program was effective in increasing the participants' responding and consequently their self-regulated stimulation input. Half of the participants showed a significant increase of both responses available from the first intervention phase. Other participants seemed to focus more on one of the two responses. Even so, they tended to have occasionally high performance frequencies also with regard to their non-dominant (not significantly increased) response. Finally, all participants showed clear signs of satisfaction/happiness during the intervention sessions.

**Conclusions:**

The program represents a potentially useful approach for enabling people with extensive multiple disabilities to self-regulate their access to preferred environmental stimulation and improve their mood.

## Introduction

People with extensive motor or motor and sensory impairment and severe/profound intellectual disability may be unable to profitably interact with objects, to engage in functional communication with others, and to control environmental stimuli (1–5). This can lead them to a condition of isolation and detachment with a consequent reduction in their level of stimulation input and possibly a state of dissatisfaction and unhappiness (6–11). While traditional programs directed at teaching self-care and occupational skills are largely inapplicable with them due to their extensive disabilities, a number of other intervention strategies have been reported as possible means to alleviate their situation and improve their quality of life. Those strategies include, among others, increased social interaction, environmental enrichment, use of multisensory rooms, and use of technology solutions to support self-regulated stimulation (2, 12–15).

Increased social interaction is an approach that implies an effort from staff, family members and others to multiply the occasions of contact with the person with disabilities so as to provide attention and possibly communication opportunities (1, 2, 5). Environmental enrichment consists of staff ensuring and regulating the availability of various forms of stimulation within the context to increase the person's sensory input and possibly promoting the person's level of satisfaction/happiness (14, 16–19). The use of multisensory rooms is an approach designed to engage the person's senses through the presence of visual, auditory, tactile, and olfactory stimulation sources (20). These multiple forms of stimulation input are considered important to improve the person's wellbeing and overall satisfaction/happiness (12, 20, 21). The use of technology solutions to support self-regulated stimulation is an approach based on providing the person with microswitches (e.g., small object-like sensors linked to a computer) that can be activated *via* simple responses such as hand, head or finger movements (22). By activating the microswitches, the person can access brief periods of preferred environmental stimulation in an independent (self-regulated) manner (3, 4, 14, 23).

While all of the aforementioned strategies are deemed to be viable approaches to alleviate the situation of persons with motor or sensory-motor impairments and intellectual disabilities, some clarifications about their characteristics and application costs may be important. For example, the increased social interaction strategy is the only one to be largely based on a specifically human form of stimulation and thus it has the likely advantage of a direct human contact and the disadvantage of a relatively large application cost in terms of staff or family's time (5, 14). Multisensory rooms may represent the most elaborate intervention strategy and also the most costly in terms of the equipment required (12, 21). Finally, the use of basic technology solutions (e.g., microswitches linked to a computer) to provide brief stimulation periods contingent on participants' simple/small responses is the only strategy that focuses on enabling the person to have an active role in the stimulation process, that is, to self-determine/regulate their stimulation input (14, 23–25). Learning stimulation self-regulation may be relevant because it counters one of the persons' most serious problems (i.e., passivity) and builds active responding and participation. Moreover, it is likely to increase the persons' attention/involvement and stimulation enjoyment thus helping them improve their mood and quality of life (14, 24, 26–29).

Studies have been conducted that document (a) the possibility of implementing the last approach (with stimulation self-regulation) successfully and (b) the seemingly greater impact of such an approach on the persons' mood compared to the impact of approaches using externally regulated stimulation (14, 18). Typically, studies have selected one specific response of the persons involved in the intervention and ensured that such response would be followed by brief periods of stimulation at each occurrence (14, 15, 30–33). Notwithstanding the positive data available, it might be argued that the use of one specific response movement is not necessarily the most effective and economical strategy. The use of two (or perhaps even more) response movements (e.g., head and elbow movements, arm upward and arm downward movements, or movements to touch a left and a right area of the desk) may be viewed as a desirable alternative for two reasons. First, using two response movements as means to access stimulation (a) may lead to an increased number of stimulation occasions particularly at the beginning of the intervention (when the level of any specific response is still low) and thus (b) may foster the persons' alertness, attention and motivation to be active (33). Second, some of these persons may find the level of comfortableness of a specific (selected) response movement to change across periods of the day or across days (e.g., due to slight changes in the persons' position and/or variations in their neurophysiological condition) (34, 35). The possibility of using two response movements to access stimulation would allow the persons to rely more heavily on the more comfortable response movement at any specific time.

This study was aimed at determining whether a fairly simple technology-aided program (i.e., a program involving the use of microswitches linked to a smartphone) could be set up to enable persons with motor and sensory impairments combined with intellectual disabilities to control stimulation through two different response movements and thus manage stimulation access efficiently/comfortably. The study was also focused on (a) determining how the persons used the response movements over time (e.g., whether they had significant increases of both response movements and/or showed response variability across sessions and over time) and (b) verifying whether the persons showed signs of satisfaction/happiness during the study sessions, that is, in connection with their opportunities to control stimulation through their response movements (14). Ten persons participated in the study.

## Methods

### Participants

Table 1 lists the 10 participants (three women and seven men) by their pseudonyms and reports their chronological age, their age equivalents for Daily Living Skills (personal sub-domain) as measured *via* the second edition of the Vineland Adaptive Behavior Scales (36, 37), and the position of the microswitches used to enable their response movements to activate stimulation events. The chronological age ranged from 13 (Trudy) to 45 (Richard) years. The Vineland age equivalents were above 1 year only for four participants (i.e., Trudy, Martin, Daniel, and Dustin). All participants had severe motor impairments and were unable to ambulate. Moreover, they presented with blindness or could simply discriminate between light and darkness. One of them (Dustin) was also diagnosed with hearing loss. They attended care and rehabilitation centers for persons with intellectual and multiple disabilities. While no formal tests could be used to assess the participants' specific functioning, the psychological services of those centers had estimated (following repeated behavioral observations) their level of intellectual disability to be in the profound range.

**Table 1:** Participants' pseudonyms, chronological age, Vineland age equivalents for daily living skills (personal sub-domain), and position of the microswitches.

**Participants (pseudonyms)**	**Chronological age (years)**	**Vineland age equivalents^a^, ^b^**	**Position of the microswitches**
Dorine	26	< 1; 0	On the wheelchair: one microswitch on the headrest and one on the armrest
Trudy	13	1; 4	On the desk: one microswitch in front of the participant and one to her side
William	31	< 1: 0	On the participant's body: one microswitch on the chest and one on the hip
Thomas	35	< 1; 0	One microswitch on the participant's wrist and one on the desk in front of the participant
Alec	34	< 1; 0	A combination of two microswitches on the desk in front of the participant and a combination of two microswitches under the desk (i.e., in front of his legs)
Richard	45	< 1; 0	On the desk: one microswitch on the far right corner of the desk and one on the center/left side of it
Martin	38	1; 10	On the desk: one microswitch on the right side of the desk and one on the center/left side of it
Lilian	36	< 1; 0	On the desk: one microswitch in front of the participant and one in the front/right side of the desk
Daniel	31	1; 5	On the desk: one microswitch on the right side of the desk and one on the center/left side of it
Dustin	24	1; 8	On the desk: one microswitch at the near center-left area of the desk and one at the right side of it

a The age equivalents are based on the Italian standardization of the Vineland scales (36).

b The Vineland age equivalents are reported in years (number before the semicolon) and months (number after the semicolon).

A number of criteria were followed for the participants' inclusion in the study. First, they were largely detached and unable to access any specific environmental stimulation without staff support. Second, preliminary observations and staff reports had indicated that they had forms of apparent interest (e.g., alertness/orientation and smiles) in relation to a number of environmental stimuli. Those environmental stimuli included music and songs, family voices, and vibratory inputs. Third, they possessed response schemes (e.g., arm/hand, head, or leg movements) that could be adequate for activating the micoswitches used during the study (see Technology system) and thus could be instrumental to independently trigger brief stimulation events. Fourth, staff (a) supported an intervention program aimed at helping the participants increase their stimulation input through a self-regulated process (i.e., a process that would keep the participants positively engaged and possibly promote their initiative/self-determination and satisfaction), and (b) had approved the study and the technology used for it, which had been described and shown to them in advance.

### Ethical approval and informed consent

Given the participants' level of intellectual disability and their consequent inability to get information about and provide consent for the study, their legal representatives were called to deputize for them. That is, the legal representatives, who had received detailed information about the study, were asked to read and sign a consent form on behalf of the participants. The study complied with the 1964 Helsinki declaration and its later amendments and was approved (including the aforementioned consent process) by an institutional Ethics Committee.

### Setting, research assistants, sessions, and stimuli

Quiet areas/rooms of the care and rehabilitation facilities that the participants attended served as setting for the baseline and intervention sessions. The participants' usual areas within those facilities (e.g., occupational or living rooms) served as setting for the control sessions. Three research assistants were in charge of the study (i.e., responsible for implementing the study sessions of all participants and collecting part of the data; see below). All three were familiar with the use of technology-aided interventions with people with intellectual and multiple disabilities as well as with data collection procedures. Baseline, intervention and control sessions lasted 10 min and were implemented on an individual basis, typically two to four times a day, 3–6 days a week (in line with participants' availability).

The stimuli used during the intervention sessions included a variety of songs and other musical pieces as well as staff and family members' voices or vibratory inputs in different parts of the body. The stimuli, which had been recommended by staff, were selected for the study after a preference screening procedure. The screening procedure involved the presentation of two or three segments of each of the song, music, voice stimuli assessed for the participant for about 10 non-consecutive times over several assessment instances (4, 38). The only exception occurred for Dustin, for whom the aforementioned (auditory) stimuli were replaced by vibratory stimuli presented in each of two or three preselected parts of the body. A stimulus was retained for use during the intervention sessions if the research assistant and staff member involved in the screening procedure concurred in reporting that the participant had positive reactions (e.g., orientation or indices of satisfaction/happiness; see below) during about or more than 50% of the presentations.

### Technology system

The technology system used during the intervention sessions involved a Samsung Galaxy smartphone with Android operating system combined with a Bluetooth Encore Plus interface (leonardoausili.com), which was linked to two microswitches (or two pairs/combinations of microswitches for Alec; see Table 1). For Dustin, the technology also included vibratory devices linked to the smartphone *via* Bluetooth. The smartphone was fitted with the Encore Plus application, which served to connect it with the microswitches and the MacroDroid application. The latter application served to program the smartphone for recording responses (throughout the study) and delivering stimulation (during the intervention phases) in line with the intervention conditions. The smartphone was also supplied with a variety of audio files representing the preferred stimulation events (i.e., music and songs which could be combined with familiar voices) for all participants except Dustin who received vibratory stimulation.

The microswitches included (a) pressure devices (i.e., small and big Buddy buttons with diameters of 6.3 and 11.5 cm, respectively; leonardoausili.com), (b) touch devices (i.e., small or big Pal Pad devices with sides measuring 10 × 6 cm and 15 × 11 cm, respectively; leonardoausili.com), and (c) proximity devices (i.e., Little Candy Corn, a triangle with a 5-cm side; leonardoausili.com). Nine participants had two microswitches on their desk, on their body, or on their wheelchair (see Table 1). The tenth participant (Alec) had a combination of two microswitches on the desk and a combination of two microswitches on a band under the desk (i.e., in front of his legs). The microswitches available on the participants' desk were embedded in a polystyrene basis fixed onto the desktop, and covered *via* a thin plastic sheet to avoid that they could be inappropriately used or damaged.

During the baseline phases, the technology system recorded the participants' responses but did not provide any stimulation for those responses. During the intervention phases, the technology system recorded the responses and delivered stimulation contingent on each of them (i.e., 10 s of preferred music/songs with or without familiar voices or 10 s of vibratory stimulation occurring alternatively in two separate parts of the body for Dustin). A response on either microswitch was ignored if emitted while the participant was receiving stimulation following a previous response. Thus, a new response was recorded only if it occurred after an interval of 10 s or more from a previous response (30). For consistency reasons, the same recording rule (i.e., a new response was to be separated from the previous by at least 10 s) was also used during the baseline (4).

### Measures and data recording

The measures involved the participants' (a) response frequencies (i.e., responses in relation to the single microswitches or combinations of microswitches for Alec) during the different phases of the study and (b) indices of satisfaction/happiness during 20–38 intervention sessions and as many control sessions, which were paired to the intervention sessions (see below). The response frequencies were recorded separately for the first response (i.e., response activating the first microswitch or combination of microswitches) and the second response (i.e., response activating the second microswitch or combination of microswitches) through the MacroDroid system log available in the smartphone. This log provided an objective and permanent data record that the research assistants used at the end of the single sessions. The indices of participants' satisfaction/happiness (a) included smiles, vocalizations, and excited body movements [i.e., behaviors that staff, families and preliminary research assistants' observations had indicated to be signs of enjoyment/pleasure (14, 18)], and (b) were recorded by the research assistants according to a partial interval system, in which 10-s observation periods were followed by 5-s recording periods (39). Interrater agreement on recording indices of satisfaction/happiness was assessed by having a reliability observer join the research assistants in data collection over 30% of the sessions in which such measure was recorded. Agreement was computed for the single sessions (by dividing the number of intervals with the same “positive” or “negative” scoring by the total number of intervals and multiplying by 100%). The session percentages ranged between 78 and 100. The single participants' means exceeded 90 and the overall mean exceeded 95.

### Experimental conditions

For each participant, baseline and intervention conditions were implemented according to an ABAB design (40). The first baseline (A) phase was preceded by an observation period aimed at identifying the types of response movements and microswitch positions suitable for the participants and thus usable during the study phases. Such a period included between 4 and 12 sessions depending on the difficulty of identifying those response movements and positions. During 20–38 sessions of the second intervention (B) phase, recording also concerned the participants' indices of satisfaction/happiness (see Measures and data recording). Each of these sessions was preceded by a control session in which the same indices were recorded under daily conditions (see below). A study coordinator, who had access to video recordings of baseline, intervention and control sessions, provided regular feedback to the research assistants about their performance during those sessions (i.e., about their implementation of procedural conditions) so as to ensure procedural fidelity (41).

### Baseline phases

During the sessions of each baseline phase, the participants sat in their wheelchair and had a desk in front of them. The only exceptions (i.e., with no desk in front of them) were Dorine and William. The microswitches were arranged on the participant's body, on the wheelchair, on the desk, or on the desk and under the desk (see Table 1). The smartphone was affixed to the desk or the wheelchair. The participants' responses were recorded but no stimulation was available for them.

### Intervention phases

During the sessions of each intervention phase, conditions differed from those used in baseline in that the technology system delivered 10 s of preferred stimulation contingent on each response occurrence (see Technology system). The stimulation consisted of 10 s of preferred music/songs with or without the superimposition of preferred familiar voices except for Dustin. Dustin, who had hearing loss, received 10 s of vibratory stimulation provided alternatively in two separate parts of the body (e.g., belly and leg) *via* two vibratory devices placed on those parts. Data recording concerned (a) the frequency of responses throughout all intervention sessions and (b) indices of participants' satisfaction/happiness during 20–38 sessions spread over the second intervention phase.

### Control sessions

Each control session (a) was paired to one of the intervention sessions in which indices of satisfaction/happiness were recorded, and (b) was carried out closely before that intervention session. During the control sessions, the participants were in their regular contexts (e.g., within the occupational or living room that they usually attended), did not have the technology system, and did not receive any specific stimulation except for possible environmental sounds and voices occurring in the contexts.

### Data reporting and analyses

The range and mean frequency for each of the two responses available for every participant are displayed in graphic form together with the range and mean of the response total (i.e., the sum of the two responses) for the different phases of the study. The range and mean percentages of intervals with indices of satisfaction/happiness across the intervention sessions and the paired control sessions are reported in table form. The differences between the response frequencies of each baseline phase and those of the following intervention phase of every participant were assessed through the Kolmogorov-Smirnov test (42). Paired *t*-tests were used for the single participants to assess the differences between their indices of satisfaction/happiness during the intervention sessions and the paired control sessions (43).

## Results

**Figure 1:**
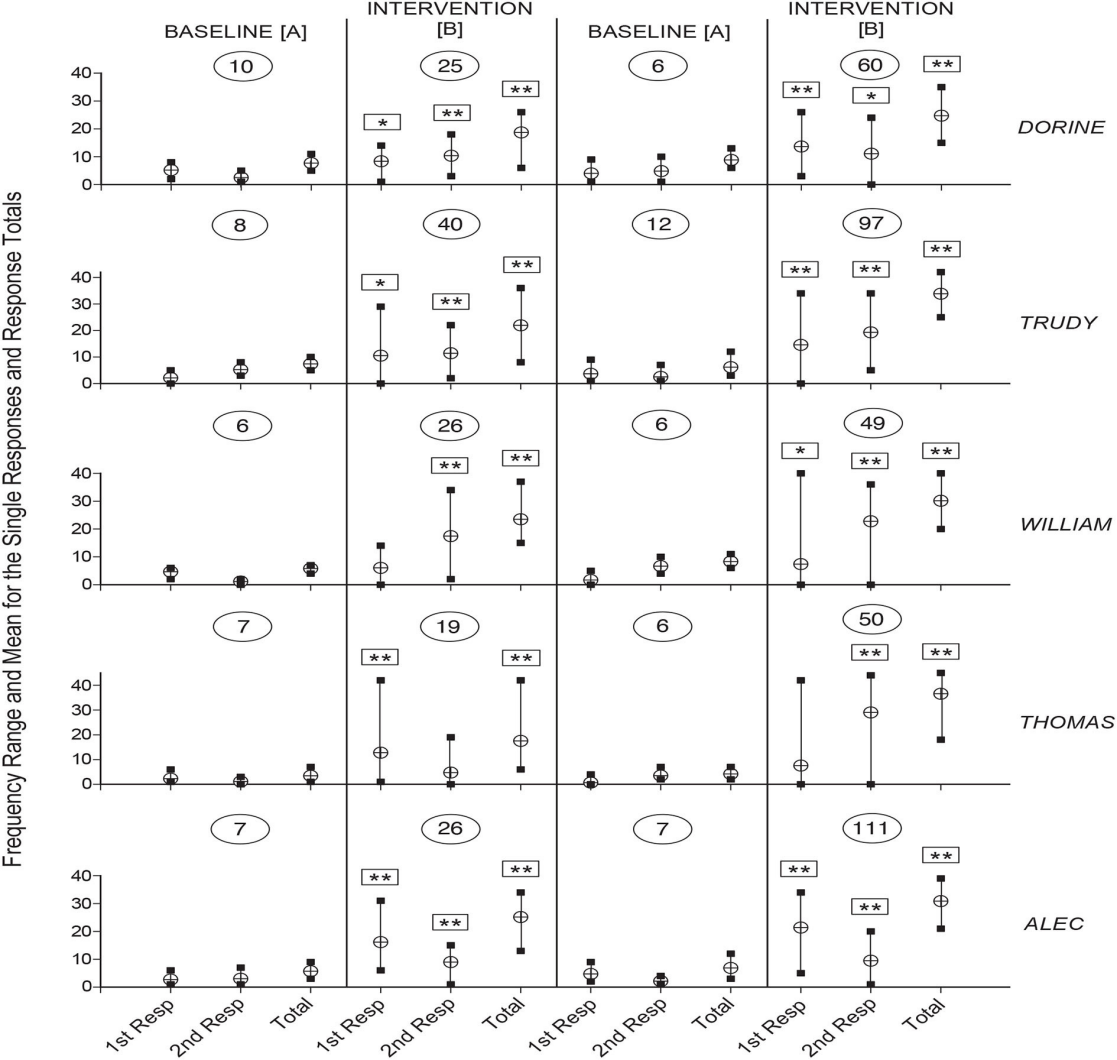
The five panels summarize the data for Dorine, Trudy, William, Thomas, and Alec. For each participant, the vertical lines with edges marked by black squares indicate the frequency range for the single responses and the response totals during the different phases of the study. The circles with the horizontal line indicate the mean frequency value for each of the response ranges. Boxes with one or two asterisks over the single responses or response totals' frequency values of the intervention phases indicate that those values differed significantly (with *p* < 0.05 and 0.01, respectively, at the Kolmogorov-Smirnov test) from the corresponding values of the previous baseline phase. The numerals within the ovals indicate the number of sessions available for the different phases of the study.

**Figure 2:**
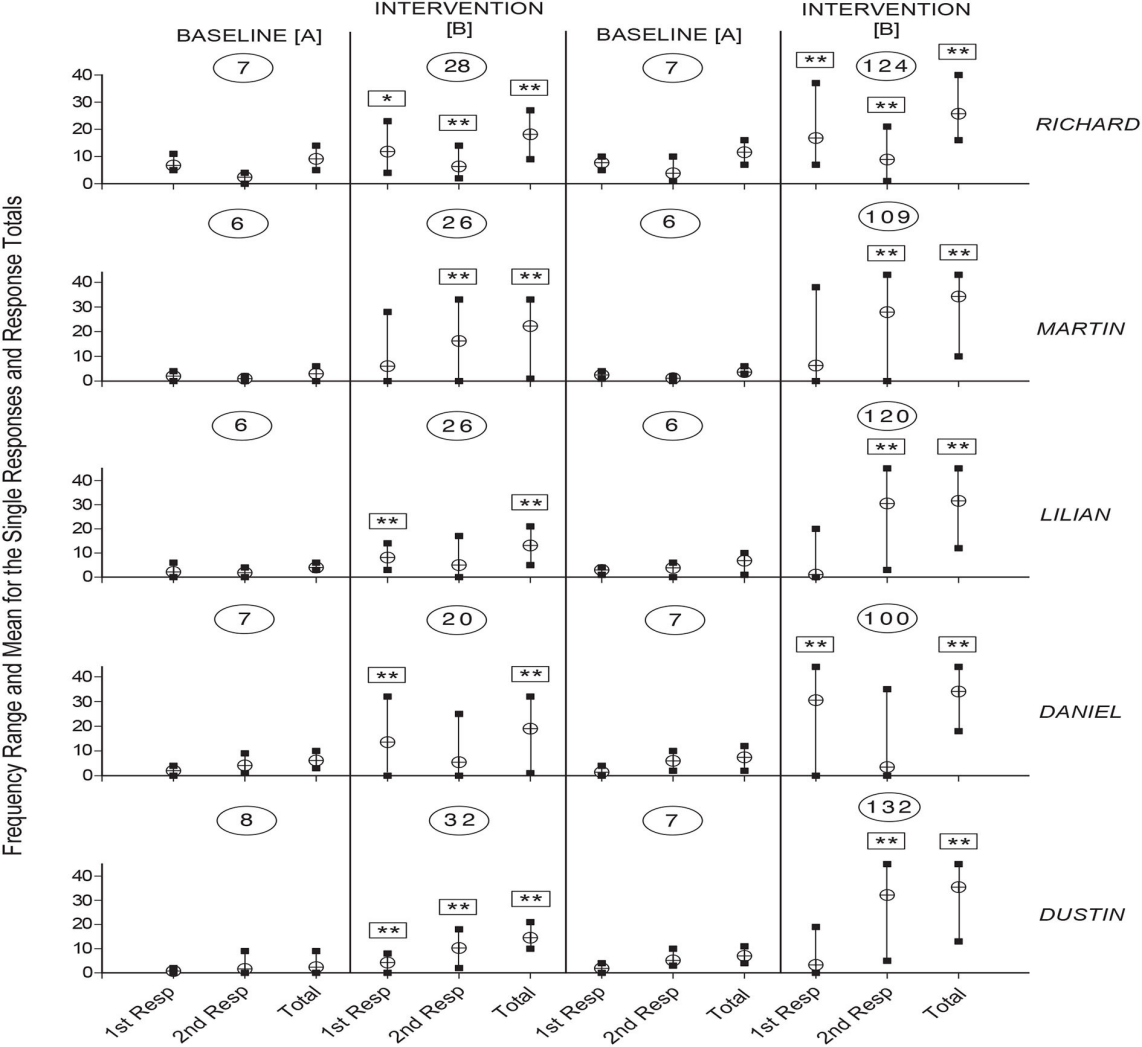
The five panels summarize the data for Richard, Martin, Lilian, Daniel, and Dustin, respectively. The data are plotted as in Figure 1.

Figures 1, 2 summarize the response data for Dorine, Trudy, William, Thomas, and Alec and for Richard, Martin, Lilian, Daniel, and Dustin, respectively. For each participant, the vertical lines with edges marked by black squares indicate the frequency range for the single responses and the response totals during the different phases of the study. The circles with the horizontal line indicate the mean frequency value for each of the response ranges. Boxes with one or two asterisks over the response frequency values of the intervention phases indicate that those values differed significantly (with *p* < 0.05 and 0.01, respectively, at the Kolmogorov-Smirnov test) from the corresponding values of the previous baseline phase. The numerals within the ovals indicate the number of sessions available for the different phases of the study.

As shown in the figures, the number of sessions the participants received varied between 6 and 10 (first baseline), 19 and 40 (first intervention phase), 6 and 12 (second baseline), and 49 and 132 (second intervention phase). During the first baseline, the mean frequencies for the single responses ranged between below 1 (Dustin's first response) and about 6.5 (Richard's first response). During the first intervention phase, the mean frequencies increased to between about four (Dustin's first response) and 17.5 (William's second response). The intervention frequencies were significantly higher than the baseline frequencies on both the first and second response for five participants (i.e., Dorine, Trudy, Alec, Richard, and Dustin), and on one of the two responses for the other five participants. The mean response totals (i.e., mean frequencies for the two responses combined) varied between about 2.5 (Dustin) and 9 (Richard) during the baseline and between about 13 (Lilian) and 25 (Alec) during the intervention phase. The intervention totals were significantly higher than the baseline totals for all participants.

The participants' mean frequency values for the second baseline were similar to those observed during the first baseline (i.e., from below 1 to about 7.5 on the single responses and between about 3.5 and 11.5 on the response totals). Their mean frequency values for the second intervention phase varied between about 3.5 and 32 on the single responses and between about 24.5 and 35.5 on the response totals. The intervention values were significantly higher than the baseline values (a) on both responses for five participants (i.e., Dorine, Trudy, William, Alec, and Richard) and one of the two responses for the other five participants, and (b) on the response totals for all participants (See Figures 1, 2).

Table 2 summarizes the participants' data concerning indices of satisfaction/happiness during the 20–38 intervention sessions in which those indices were recorded and in the 20–38 control sessions paired to the intervention sessions. The table does not include Richard as data for him were recorded only in few sessions due to a combination of practical/organizational problems and a relatively short duration of the second intervention phase. The table shows that all nine participants, for whom sufficient data were collected, had higher levels of indices of satisfaction/happiness during the intervention sessions as compared to the paired control sessions. The paired *t*-tests used to compare the intervention and control data indicate that the difference between the two sets of data was significant for each participant, with t values ranging between 3.51 and 33.59, and *p* values < 0.01.

**Table 2:** Percentage range and mean of intervals with indices of satisfaction/happiness during the intervention and control sessions and *p*-values for the paired *t*-tests comparing the two sets of data.

**Participants (pseudonyms)**	**Number of sessions**	**Intervention**		**Control**		**Paired**
		**% Range**	**% Mean**	**% Range**	**% Mean**	***t*** **- tests**
						***p <***
Dorine	30	5–60	32.42	0–42.5	6.83	0.01
Trudy	30	0–75	37.58	0–27.5	7.67	0.01
Thomas	20	47.5–80	65.5	0–5	0.50	0.01
Alec	26	37.5–70	51.63	0–2.5	0.29	0.01
Richard	38	0–65	29.41	0–20	8.75	0.01
Martin	30	0–65	43.58	0–25	9.0	0.01
Lilian	30	0–32.5	8.42	0–17.5	2.92	0.01
Daniel	30	0–75	21.0	0–30	6.83	0.01
Dustin	30	0–30	6.58	0–7.5	0.83	0.01

## Discussion

The results suggest that a relatively simple technology-aided program, which allowed participants with motor, sensory and intellectual disabilities to control environmental stimulation through two different response movements, was effective in increasing the participants' responding and consequently their self-regulated stimulation input. Several participants seemed to consistently use each of the two responses available (i.e., displaying a significant increase of both) from the first intervention phase. Other participants seemed to focus more on one of the two responses, but even so, they tended to have occasionally high performance frequencies also with regard to the non-dominant (not significantly increased) response. Finally, all participants showed clear signs of satisfaction/happiness during the intervention sessions suggesting that the program may have a positive impact on mood and quality of life (24, 27, 28, 44–46). In light of the above, a number of considerations may be in order.

First, the program used in this study relies on commercial, easily accessible technology and that makes it a reasonably usable tool for daily contexts such as care and rehabilitation facilities (47–50). Indeed, the smartphone, the microswitches, and the Bluetooth interface to link the microswitches to the smartphone are all available as mainstream devices or educational material. Similarly, the MacroDroid application is easily accessible. The overall cost of the aforementioned technology components may be ~US $550. This includes about $200 for the smartphone, about $200 for the Bluetooth interface, and an average of about $150 for two microswtiches. This cost is not irrelevant for many daily contexts. Yet, one may still consider it justifiable given the overall simplicity and limited application time demands of the program and the possibility of using it for more than one participant within the context.

Second, the fact that a number of participants showed a significant increase of each of the two responses available is noteworthy from a technical and practical standpoint. Technically, such an increase could be considered a clear indication that having two microswitches (as opposed to one) can help strengthening different response schemes and facilitating access to environmental stimulation (33). Practically, facilitating access to stimulation (by allowing participants to use different response movements to activate it) can be important to increase the participants' alertness and motivation to be responsive particularly in the early stages of the intervention [i.e., when the participants go through the process of learning to use their responding as a functional tool (39, 51)].

Third, the fact that participants who had significant increases of only one of the two responses tended to display occasionally high frequencies also for the non-dominant response (i.e., the response for which the baseline and intervention levels did not reach a statistically significant difference) may be a relevant sign. Indeed, these occasionally high frequencies may suggest that the non-dominant response played a critical role for the participant's access to stimulation during specific sessions and/or days. It may be reasonable to assume that, due to positioning or other neurophysiological conditions, the participants found such response more comfortable and convenient than the dominant response during those sessions/days and thus resorted to its use to access stimulation (34, 35).

Fourth, an intervention program that allows participants to access stimulation through the two target responses or the more convenient/comfortable response at any time may be viewed as user-friendly and have a positive impact on response performance, mood and eventually quality of life (14, 23, 26–28, 30). Moreover, a program (and the related technology system) designed to favorably match participants' skills and needs (i.e., to be user-friendly) might have a higher probability of being used over time and a lower risk of being rapidly abandoned (50, 52).

Fifth, the presence of significantly higher indices of satisfaction/happiness during the intervention sessions compared to the control sessions underlines the importance of the program for improving the participants' mood and quality of life while increasing and strengthening their responding and stimulation control (14, 30, 53–56). One might argue here that the mood improvement observed during the intervention sessions was probably not only due to the stimulation available but also to the fact that the participants could self-regulate it. While this viewpoint does not have any direct evidence in the present study (i.e., as this study did not investigate the specific role of stimulation self-regulation), it can find such evidence in previous studies comparing the impact of self-regulated vs. staff-regulated stimulation (14, 18, 57).

## Limitations and future research

Two limitations of the study may be pointed out. The first limitation concerns the fact that no direct comparison was made of the present program allowing the participants to use two responses and a conventional program relying on a single response. While it seems reasonable to believe that the present program has clear advantages over a program using a single microswitch/response, a direct comparison between the two may still be required. To carry out such a comparison, one might try to (a) expose the participants to the two programs according to a cross-over design (i.e., alternating the programs' sequence among participants) or (b) contrast the performance of a group of participants using two microswitches with the performance of a group of participants using one microswitch (43, 58).

The second limitation concerns the absence of a social validation of the program used. Such validation could be carried out through interviews of staff personnel with experience in this area (i.e., working with people with severe multiple disabilities). The interview would be preceded by a description of the program followed by the presentation of video clips showing intervention sessions, in which the program is being used with people with extensive multiple disabilities. The staff involved in the interview would be asked to rate the program in terms of its suitability to participants with extensive multiple disabilities, its impact on the participants' overall responding, physical involvement and mood, and its applicability and acceptability in daily contexts (59, 60).

In conclusion, the results suggest that a technology-aided program allowing the use of two different responses to access preferred environmental stimulation was effective in helping people with extensive multiple disabilities to (a) increase their responding, thus increasing their self-determined/regulated stimulation input, and (b) boost their level of satisfaction/happiness with a consequent enhancement of their quality of life. While the results are encouraging, one cannot make general statements about the program's positive impact, advantages, and usability in daily contexts until new research has addressed the limitations of this study. New research may also seek to upgrade the program to foster its effectiveness and promote its use with participants with different characteristics.

## Data availability statement

The original contributions presented in the study are publicly available. This data can be found here: https://osf.io/qj3z4/.

## Ethics statement

The study was reviewed and approved by the Ethics Committee of the Lega del Filo D'Oro, Osimo, Italy. Written informed consent to participate in this study was provided by the participants' legal guardian/next of kin.

## Author contributions

GL was responsible for setting up the study, acquiring and analyzing the data, and writing the manuscript. NS, MO'R, and JS collaborated in setting up the study, analyzing the data, and writing/editing the manuscript. GA, VC, and LD contributed in evaluating and arranging the technological aspects of the study, acquiring and analyzing the data, and editing the manuscript. All authors contributed to the article and approved the submitted version.
